# Effect of *Aloe vera* and clove powder supplementation on carcass characteristics, composition and serum enzymes of Japanese quails

**DOI:** 10.14202/vetworld.2015.664-668

**Published:** 2015-05-27

**Authors:** Hujaz Tariq, P. V. Raman Rao, Rita S. Raghuvanshi, B. C. Mondal, S. K. Singh

**Affiliations:** 1 Department of Animal Nutrition, College of Veterinary and Animal Sciences, Govind Ballabh Pant University of Agriculture and Technology, Pantnagar, India; 2 Dean Home Science, Govind Ballabh Pant University of Agriculture and Technology, Pantnagar, India; 3 Department of Livestock Production and Management, College of Veterinary and Animal Sciences, Govind Ballabh Pant University of Agriculture and Technology, Pantnagar, India

**Keywords:** *Aloe vera*, ALT, AST, clove, dressing percentage, giblet, Japanese quails

## Abstract

**Aim:**

The aim was to study the effect of *Aloe vera* and clove powder supplementation on carcass characteristics, composition and serum enzymes of Japanese quails.

**Materials and Methods:**

The study was conducted on 120-day-old Japanese quails, which were randomly divided into four treatment groups *viz*. T_1_-control, T_2_-0.5% *A. vera*, T_3_-0.5% clove, and T_4_-(0.25% *A. vera* + 0.25% clove) powder, each having three replications consisting of ten Japanese quails. The birds in control group (T_1_) were fed no supplement whereas in treatments T_2_-T_4_ birds were supplemented with *A. vera* leaf powder, clove, and mixture of both (powdered form) at 0.5, 0.5, and 0.25+0.25% of feed, respectively. After 7 days of brooding, a feeding trial of 35 days was conducted. On 35^th^ day of trial birds were sacrificed and dressing percentage, cut up parts, organ weight, processing losses and carcass meat composition of the breast, thigh, and drumstick were recorded. Blood samples were collected on the same day and serum was separated to evaluate serum enzymes alanine aminotransferase (ALT) and aspartate aminotransferase (AST).

**Results:**

It showed significantly (p<0.05) higher values of dressing percentage with and without giblet and breast weight in the T_2_-T_4_ group as compared to the T_1_ group. No significant (p>0.05) differences were observed in giblet weight, other cut up parts and composition of the breast, thigh, and drumstick muscles in all the treatment groups. Serum ALT and AST did not vary significantly among different treatment groups.

**Conclusion:**

*A. vera* and clove supplementation improved the dressing percentage and breast weight without adversely affecting the meat composition and serum enzymes. Thus, these can be used as a growth promoter in Japanese quails.

## Introduction

The fast growing nature of broilers and their short generation interval have been associated over the years with the use of antibiotic growth promoters. Antibiotics improve growth performance mainly by decreasing some pathogenic and increasing useful microorganisms of intestinal micro flora. European Union has banned the use of these antibiotics in poultry due to their contribution to the development of resistance, which negatively impacts human health. However, there is a demand to enhance both quantity and quality, i.e., low fat and antibiotics free edible meat for human consumption. In the last couple of decades, Japanese quails have provided a great potential in this regard due to its inherent ability to produce low-fat meat. Recently, research is also being carried to replace these antibiotics with natural feed additives in poultry feeds [1]. Spices, herbs, medicinal plants, and organic acids have been successfully used as growth promoters in the poultry diets [2,3].

*Aloe vera* is a unique plant, which is having great medicinal value [4]. It has antibacterial activity against various pathogenic bacteria, mainly Gram-positive bacteria which make it fit to be used for medication, food, and cosmetic purposes [5]. Many studies have shown antibacterial, antiseptic, anti-inflammatory, and immune modulator effect of *A. vera* [6-8]. On the other hand, clove and its essential oil have also been found as effective growth promoters in broilers [9], which mainly act by decreasing intestinal pathogens [10]. In addition antiseptic, appetite and digestion stimulant [11], anti-inflammatory and antioxidant [12] activities of clove and its ingredients have been reported. Based on the above characteristics we hypothesized that *A. vera* and clove supplementation can be effective to improve carcass characteristics of Japanese quails without affecting carcass composition and serum enzymes. Thus, the present study was conducted to study the effect of *A. vera* and clove powder supplementation on carcass characteristics, composition, and some serum enzymes of Japanese quail.

## Materials and Methods

### Ethical approval

The protocol and experimental design of the study were approved by the Institutional Ethics Committee, College of Veterinary Science & Animal Husbandry, Govind Ballabh Pant University of Agriculture & Technology (GBPUAT), Pantnagar, Uttarakhand, India.

### Preparation of *A. vera* and clove powder

*A. vera* leaves were collected from “Medicinal Plants Research and Development Centre, GBPUAT, Pantnagar, India” and clove buds were purchased from the market. Green *A. vera* leaves were initially sun dried for 3-4 days to a moisture content of approximately 70%. After this *A. vera*, as well as clove buds, were dried in an oven at 60°C up to moisture content level below 10%. Then both leaves and clove buds were crushed in a grinder to make it fine. It was passed through a fine meshed wire sieve to obtain a uniform powder.

### Birds and experimental design

An experiment was conducted on 120-day-old Japanese quail chicks (*Coturnix coturnix japonica*) each weighing 6-8 g, belonging to the same hatch, which were reared at Instructional Poultry Farm, GBPUAT, Pantnagar, India. After 1 week of brooding period, chicks were used for feeding trial. After weighing the chicks individually, birds were randomly allocated into four different treatment groups with three replicates of ten quails in each pen. The chicks used for the experiment were housed in deep litter system with partitions of one square inch wire mesh. The housing and managemental conditions were similar for all groups of birds in the trial. Standard experimental broiler feed was fed as per the specifications of Bureau of Indian Standards [13]. The birds in control group (T_1_) were given no supplement, whereas in treatments T_2_-T_4_ birds were supplemented with *A. vera* leaf powder, clove, and mixture of both (powdered form) at 5, 5, and 2.5 + 2.5 g/kg of feed, respectively.

### Carcass characteristics and composition

At the end of feeding trial, i.e., on 35^th^ day of feeding trial, two birds (1 male + 1 female) from each replicate (6 birds/treatment) were randomly selected and slaughtered for carcass trait study as per the standard procedure. Prior to slaughter, the Japanese quails were off fed for 12 h. Carcass traits *viz*. dressing percentage with or without giblet, cut-up parts (drumstick, thigh, wing, neck, back, and breast), organ weights (liver, heart, and gizzard), and processing losses (head, shank, blood, and feather) were analyzed on % live body weight basis. The meat samples were collected from the breast, thigh, and drumstick separately from each replicate and were analyzed for proximate composition [14].

### Serum enzymes

Blood samples were collected from two birds from each replicate at the end (35^th^) day of feeding trial. Blood was collected aseptically from the wing vein in sterilized disposable syringes (24 gauge needle) and serum was separated to study serum alanine aminotransferase (ALT) and aspartate aminotransferase (AST) in IU/L using kits (Span Diagnostics Ltd.).

### Statistical analysis

Statistical analysis was done using completely randomized design, one-way classification as per the procedure given by Snedecor and Cochran [15]. Significant differences among different treatments were identified using Duncan’s Multiple Range Test and a p<0.05 was considered to be statistically significant.

## Results and Discussion

### Carcass characteristics

The effects of *A. vera* and clove powder supplementation on dressed yield, organ weight, cut up parts, and processing losses of Japanese quails have been presented in Tables-1 and 2. Result showed an increase (p<0.5) in dressing yield with and without giblet in T_2_-T_4_ as compared to control. These results are in comparison with Amaechi and Iheanetu [16] who found higher (p<0.5) dressing percentage in broilers supplemented with 2% *A. vera* than the control group. Simsek *et al*. [17] also found that adding plant extract thyme, clove, and anise had a positive effect on the carcass yield. The overall means for breast weight (% live body weight) showed an increase (p<0.5) in T_2_-T_4_ with respect to T_1_ but no significant difference was found between T_2_-T_4_. It is similar to Doley *et al*. [18] who found increased (p<0.05) weight of breast, thigh, and leg in groups supplemented with 0.5% Yeast and *A. vera* as compared to control group. Increased breast weight was also found in broilers supplemented with clove and cinnamon [19]. Our study showed non-significant effect of *A. vera* and clove supplementation on other cut up parts (thigh, back, neck, wing, and drumstick), organ weight (heart, liver, and gizzard), and processing losses (head, neck, shank, and feather) on % live body weight basis of Japanese quails. It is similar to findings of Mehala and Moorthy [20] and Dalkiliç and Guller [21] who reported non-significant difference in thigh, neck, back, wing, liver, heart, and gizzard weight when supplemented with *A. vera* and clove, respectively in broilers. Thus, in present study supplementation of *A. vera* and clove showed increased dressing percentage and breast weight without any effect on other cut up parts and organ weight, which suggests a better growth in treatment groups. The better growth rate of *A. vera* and clove could be due to acemannan - a mannose polymer [22], and eugenol present in them respectively. These have been found to increase the population of beneficial microorganisms like lactobacillus [23,24] and decrease the harmful bacteria like *Escherichia coli* in intestine [10]. This decrease in pathogens could be due to binding of mannan oligosaccharide with mannose-specific Type 1 fimbriae of pathogens which prevents their colonization at intestinal epithelium, resulting their excretion from intestine [25]. The improved microbial health in turn results in better nutrient utilization and growth in poultry [26].

**Table-1:** Effect of *A. vera* and clove powder supplementation on carcass yield, cut up parts, and organ weight (% live weight) of Japanese quails.

Treatments	T_1_	T_2_	T_3_	T_4_
Carcass yield (%)				
Dressed yield with giblet	71.07^b^±0.33	73.61^a^±0.29	73.99^a^±0.41	73.49^a^±0.38
Dressed yield without giblet	65.51^b^±0.26	68.62^a^±0.47	68.43^a^±0.57	67.57^a^±0.65
Cut up parts (% live weight)				
Thigh	10.02±0.18	10.73±0.22	10.32±0.25	10.64±0.14
Breast	22.32^b^±0.63	24.59^a^±0.22	24.49^a^±0.20	23.99^a^±0.70
Drumstick	7.60±0.01	8.64±0.03	8.32±0.08	8.25±0.06
Back	19.68±0.42	19.15±0.30	19.27±0.08	19.47±0.09
Neck	3.23±0.16	3.36±0.03	3.23±0.08	3.44±0.06
Wing	5.58±0.10	5.78±0.16	5.52±0.32	5.80±0.38
Organ weight (% live weight)				
Heart	0.65±0.01	0.67±0.01	0.69±0.01	0.69±0.01
Liver	2.18±0.04	2.29±0.04	2.20±0.11	2.24±0.03
Gizzard	2.77±20	2.32±04	2.55±23	2.61±02

Values with different superscripts column-wise differ significantly (p<0.05), *A. vera=Aloe vera*

**Table-2:** Effect of *A. vera* and clove supplementation on processing losses (% live weight) of Japanese quails.

Treatments	Processing losses (% live weight)			

	Blood loss	Feather loss	Head	Shank
T_1_	3.40±0.06	8.28±0.09	4.36±0.10	2.13±0.05
T_2_	3.63±0.02	8.59±0.23	4.310±0.17	2.15±0.01
T_3_	3.50±0.10	8.30±0.07	4.57±0.07	2.13±0.05
T_4_	3.43±0.10	8.46±0.13	4.49±0.21	2.14±0.03

A.vera=Aloe vera

### Carcass composition

The proximate composition with respect to moisture %, crude protein (CP), ether extract (EE), and total ash contents in drumstick, thigh, and breast muscle samples have been presented in Table-3. Results showed numerically higher CP and lower EE in drumstick meat in comparison to breast and thigh meat in all treatment groups. Proximate composition with respect to moisture%, CP, EE, and total ash contents estimated in drumstick, thigh, and breast muscle did not vary due to *A. vera* and clove powder supplementation. It is contrary to Bonos *et al*. [27] who reported a decrease in ether extract in breast muscle due to mannan oligo-saccharide supplementation in Japanese quails. However, results of moisture, crude protein, and total ash content were comparable to our study. Unfortunately, little information is available on the effect of herbs on meat composition and thus more studies are required on this regard.

**Table-3:** Effect of *A. vera* and clove supplementation on the breast, thigh, and drumstick meat composition of Japanese quails.

Treatments (%)	T_1_	T_2_	T_3_	T_4_
Breast				
Moisture	71.52±0.94	71.42±0.51	71.46±1.17	72.79±0.95
CP	19.69±0.22	19.32±0.49	19.55±0.07	19.87±0.06
EE	3.21±0.04	3.28±0.06	3.06±0.02	3.16±0.14
Total Ash	1.28±0.03	1.28±0.07	1.46±0.07	1.40±0.02
Drumstick				
Moisture	73.54±1.07	73.94±0.38	73.99±0.88	72.36±1.14
CP	20.50±0.41	20.12±0.29	20.78±0.36	20.63±0.33
EE	2.38±0.05	2.29±0.02	2.35±0.06	2.21±0.09
Total Ash	1.23±0.17	1.18±0.14	1.23±0.05	1.27±0.17
Thigh				
Moisture	68.27±0.98	68.38±1.08	67.88±0.81	68.40±0.70
CP	19.77±0.09	19.57±0.21	19.46±0.16	19.53±0.15
EE	2.80±0.01	2.85±0.04	2.73±0.06	2.84±0.04
Total Ash	1.36±0.09	1.42±0.09	1.37±0.07	1.26±0.05

CP=Crude protein, EE=Ether extract, *A. vera=Aloe vera*

### Serum enzymes

The effect of *A. vera* and clove powder supplementation on serum enzymes ALT and AST have been presented in Table-4. Results showed no difference in ALT and AST between T_1_-T_4_ groups which is in agreement with Singh *et al*. [28] who reported the non-significant effect of *A. vera* supplementation in broiler chickens. Similar findings were observed by Azadegan *et al*. [29] who did not found any significant difference in serum ALT and AST in broilers supplemented with different levels of clove essential oils. This may be attributed to the fact that both *A. vera* and clove powder supplementation did not have detrimental effect in poultry.

**Table-4:** Effect of *A. vera* and clove powder supplementation on serum enzymatic profile (IU/L) of Japanese quails.

Treatment	AST (IU/L)	ALT (IU/L)
T_1_	160.23±3.11	11.72±0.34
T_2_	151.04±3.06	10.92±0.17
T_3_	157.48±3.00	11.15±0.24
T_4_	154.99±2.99	11.16±0.30

ALT=Alanine aminotransferase, AST=Aspartate aminotransferase, *A. vera=Aloe vera*

## Conclusion

From the present study, it can be concluded that both *A. vera* and clove powder supplementation can improve the carcass characteristics such as dressing percentage and breast weight of Japanese quails without any harmful effect on organ weight, carcass composition, and serum enzymes in Japanese quails. Thus, it can be concluded that both *A. vera* and clove have growth promoting characteristics and can be used as growth promoters in broilers.

## Authors’ Contributions

HT: Concept and design of the experiment along with acquisition, analysis, and interpretation of data under the direct guidance and supervision of PVRR, RSR, BCM, and SKS. All the authors read and approved the final manuscript.
